# Photocleavable Guide crRNAs for a Light-Controllable CRISPR/Cas9 System

**DOI:** 10.3390/ijms252212392

**Published:** 2024-11-19

**Authors:** Lubov Sakovina, Ivan Vokhtantsev, Elizaveta Akhmetova, Mariya Vorobyeva, Pavel Vorobjev, Dmitry O. Zharkov, Darya Novopashina

**Affiliations:** 1 Institute of Chemical Biology and Fundamental Medicine SB RAS, 630090 Novosibirsk, Russia; kodi99@list.ru (L.S.); ivanvohtancev@gmail.com (I.V.); liza.khabardina@mail.ru (E.A.); kuzn@niboch.nsc.ru (M.V.); vorobyev@niboch.nsc.ru (P.V.); dzharkov@niboch.nsc.ru (D.O.Z.); 2 Faculty of Natural Sciences, Novosibirsk State University, 630090 Novosibirsk, Russia

**Keywords:** photoregulated CRISPR/Cas9 system, photocleavable crRNA, 2′-modified crRNA, off-target effects

## Abstract

The design of controllable and precise RNA-targeted CRISPR/Cas9 (Clustered Regularly Interspaced Short Palindromic Repeats) systems is an important problem of modern molecular biology and genetic technology. Herein, we have designed a series of photocleavable guide CRISPR RNAs (crRNA) and their 2′-modified (2′-fluoro and locked nucleic acid) analogs containing one or two 1-(2-nitrophenyl)-1,2-ethanediol photolabile linkers (PL). We have demonstrated that these crRNAs can be destroyed by relatively mild UVA irradiation with the rate constants 0.24–0.77 min^−1^ and that the photocleavage markedly slows down the action of Cas9 nuclease in the model in vitro system. Two PLs provide more rapid crRNA destruction than a single linker. PLs in the crRNA structure improve the specificity of DNA cleavage by Cas9 nuclease for the fully complementary target. The application of photocleavable crRNA in CRISPR/Cas9 genome editing permits the system to be switched off in a spatiotemporally controlled manner, thus alleviating its off-target effects.

## 1. Introduction

The design of controllable molecular tools that can be switched on and off in time and space with specific signals is a hot area of modern molecular biology and biochemistry [1,2,3,4]. In particular, light is a precisely controlled trigger that can be used as a non-invasive regulator of biochemical processes and molecular genetic tools in living cells [4,5,6].

The RNA-programmed CRISPR/Cas9 system is widely used nowadays for genome editing, regulation of gene expression and other tasks requiring manipulations with genetic material [7,8,9]. Improvements in this universal tool in terms of precision and efficiency are of immense interest. The development of regulatable CRISPR/Cas9 systems, including photoregulatable ones, seems to be one promising way to control the action of genome editors in living cells [10,11,12,13,14]. The natural CRISPR/Cas9 effector complex consists of Cas9 nuclease and a pair of short RNA molecules, CRISPR RNA (crRNA) and transactivating CRISPR RNA (tracrRNA), which can be artificially fused into a single guide RNA (sgRNA) molecule [7,15]. So far, most studies devoted to the design of photoregulatable CRISPR/Cas9 systems have been concerned with photoactivation [12,13]. For example, the activity of the protein part of the complex, the Cas9 nuclease, can be controllably triggered by the introduction of a photocaged Lys residue that prevents DNA cleavage until being released by ultraviolet (UV) irradiation [16] or by photoactivatable dimerization of Cas9 fragments using a rapamycin-inducible system [17]. On the other hand, approaches to CRISPR/Cas9 control using modified nucleic acids are inherently simpler from the synthetic point of view. Usually, they involve some sort of optically induced decaging, with the introduction of photolabile links to guide the RNA itself or additional complementary oligonucleotides [12,18,19]. On the contrary, only a few works describe RNAs specifically designed for gene editing systems turned off by light [20,21]. Systems of this kind utilize photolabile linkers in the gRNA for inactivation.

Off-target DNA cleavage by Cas9 is the most common source of unwanted outcomes of genome editing [22,23,24]. One of the discussed strategies to reduce off-target effects is to minimize the time window of the presence of active Cas9/gRNA complexes in the cell [25,26]. Although the system can be switched off by chemogenetic means, such as small-molecule inhibitors, anti-CRISPR proteins, induced protein degradation, etc. [13,14], this approach is generally plagued by variable efficiency of inhibitor delivery, slow kinetics, and incomplete deactivation. Photocleavable gRNAs allowing for light-based Cas9 inactivation present a viable alternative to overcome these difficulties. Moreover, photocleavable gRNAs that can be switched off almost instantly could also be useful when only a fraction of specific DNA sequences present in the cell need to be edited or deleted, as in the therapy of diseases caused by gene duplication or multiplication. A clinically relevant example relying on this approach is the *PMP22* (Peripheral myelin protein 22) gene on chromosome 17, whose duplication causes the Charcot–Marie–Tooth type 1A (CMT1A) disease, whereas its insufficiency leads to hereditary neuropathy with liability to pressure palsies. Thus, controllable gene editing by the CRISPR/Cas9 system was proposed as a means to restore the optimal level of the expression of *PMP22* [27,28]. Given that ex vivo editing of induced pluripotent stem (iPS) cells derived from CMT1A patients and their differentiation to Schwann cells have been achieved and protocols for re-myelinating allogeneic iPS cells transplantation in humans have been established [29,30,31], photocontrollable editing of the gene copy number followed by autotransplantation could be a viable therapeutic option.

It is evident that photoregulatable gRNAs with better efficiency, stability and specificity would be a useful addition to the CRISPR/Cas9 toolbox. The aim of this present work was to design photocleavable crRNAs carrying nucleotide modifications at position 2′ (2′-fluoro or locked nucleic acids, LNA), which are often used to improve the biological properties of nucleic acids.

## 2. Results

### 2.1. Design and Synthesis of Photocleavable Guide RNA

**Figure 1:**
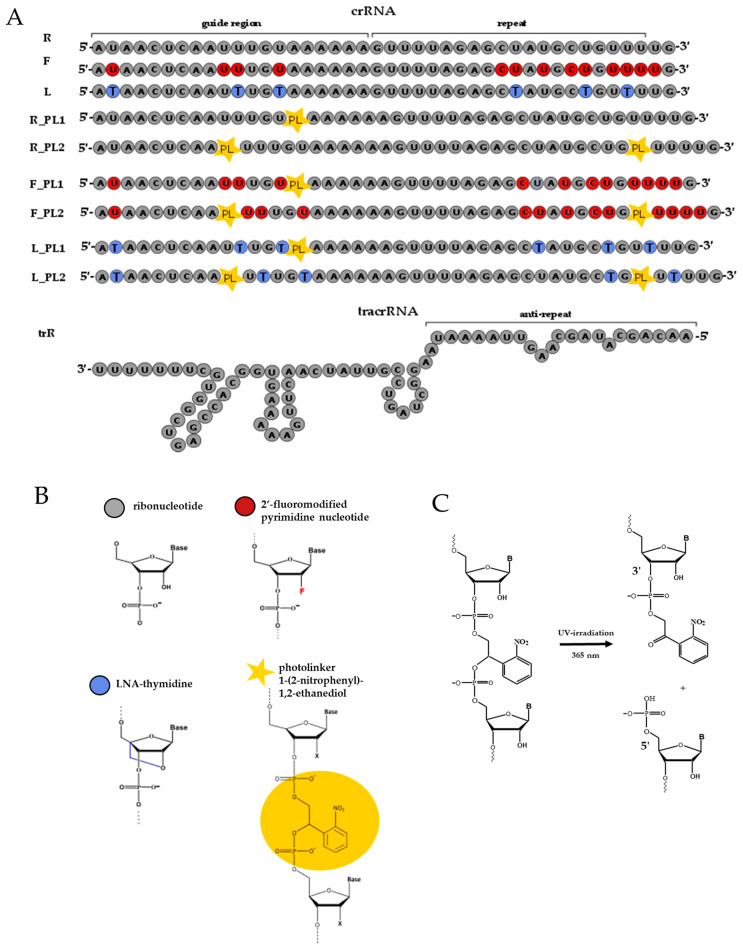
(**A**) Sequences of native (R) and 2′-modified crRNA (F, L) and their analogs with one (R_PL1, F_PL1 and L_PL1) or two (R_PL2, F_PL2 and L_PL2) photolabile linkers, and native tracrRNA (trR). (**B**) Structures of ribonucleotide, 2′-fluoro-modified nucleotide, LNA-nucleotide and photolabile linker (PL) are presented. (**C**) Reaction of photocleavage of PL in the crRNA structure.

Previously, we developed 2′-modified (2′-fluoro and LNA) RNAs as analogs of natural crRNA with enhanced nuclease stability and specificity [32]. In this present study, we designed a series of new photocleavable analogs of native, 2′-fluoro and LNA-modified crRNA for light-controlled inactivation of the CRISPR/Cas9 system. As a light-sensitive moiety, we have chosen a linker of the 1-(2-nitrophenyl)-1,2-ethanediol nature, which was previously successfully used within different photocleavable oligonucleotide constructs [33,34,35] (Figure 1A, Supplementary Table S1).

Both sgRNA and a pair of crRNA and tracrRNA can be used to guide Cas9 nuclease activity. Herein, we opted for the crRNA/tracrRNA strategy due to synthetic convenience considerations. The native (R) and modified (F, 2′-fluoro, and L, LNA) photocleavable crRNA and non-modified tracrRNA (trR) were synthesized by a solid-phase phosphoramidite method using a custom phosphoramidite monomer of 1-(2-nitrophenyl)-1,2-ethanediol [33,34]. Photocleavable crRNA contained either one or two photolabile linkers (Figure 1A, Supplementary Table S1). In the case of a single photolabile linker (R_PL1, F_PL1, L_PL1), it was placed near the crRNA seed region as this site is quite sensitive to structural changes and thus could well suit controllable deactivation. For RNA containing two PLs (R_PL2, F_PL2, L_PL2), the first was located in the non-seed region, while the second one was inserted 27 nucleotides away into the fragment that interacts with tracrRNA and the protein. Two photolinkers were placed far apart to maintain the stability of the RNA/DNA heteroduplex or RNA/RNA duplex in the context of the ribonucleoprotein complex until irradiation. We assumed that the cleavage of the first linker would destabilize the heteroduplex of crRNA with target DNA and the cleavage of the second linker would disrupt Cas9–RNA interaction. All synthesized RNAs were isolated by gel electrophoresis, and their homogeneity was analyzed by reverse-phase HPLC (High Performance Liquid Chromatography) and denaturing PAGE (Polyacrylamide Gel Electrophoresis) (Figure S1).

### 2.2. Photocleavable crRNA Degradation upon UV Irradiation

Photocleavage of the 1-(2-nitrophenyl)-1,2-ethanediol linker yielded two oligonucleotide fragments, one bearing 5′-phosphate and another containing 2-(2-nitrophenyl)-2-oxoethyl phosphate at the 3′-terminus (Figure 1C) [36]. To determine the conditions for exhaustive PL cleavage, we followed the time course of cleavage of synthesized modified crRNAs upon irradiation at 365 nm, which is optimal for PL photodegradation [37]. Water solutions of crRNA were UV-irradiated at 365 nm for up to 30 min, and the products were analyzed by reverse-phase HPLC. The peak corresponding to the initial PL-containing crRNA eventually disappeared, and peaks with lower retention times emerged, which we attributed to the products of photodegradation (Figure S2). From the integrated areas of the peaks, we calculated the photocleavage rate constants (*k*) and half-life (*t*_1/2_) values (Table 1).

As can be seen, 2′-modified crRNAs with two PLs (F_PL2, L_PL2) were degraded more quickly by irradiation than their counterparts containing a single PL (F_PL1, L_PL1). At the same time, 2′-modified crRNAs (the F and L series) were more stable than the native ones (the R series). Particularly, LNA-modified crRNAs demonstrated the longest half-life upon UV irradiation. Considering these results, for all subsequent experiments, we chose 30 min of UV irradiation time, which guaranteed >99.9% cleavage of photosensitive crRNA, even for the most stable variants.

### 2.3. Cleavage of Plasmid DNA by Cas9 in a Complex with Photocleavable crRNA and Non-Modified tracrRNA

We then investigated the influence of 2′-modifications in combination with a PL within crRNA on DNA cleavage by Cas9 nuclease. As a model DNA target, we used the pBlueScript II SK(−) plasmid containing a well-characterized Psp2 protospacer [7] and a TGG protospacer adjacent motif (PAM). Herein, we compared DNA cleavage by the Cas9/crRNA/tracrRNA ribonucleoprotein that was non-irradiated, UV-irradiated after the assembly of the complex, or assembled from Cas9, tracrRNA, and pre-irradiated crRNA (Figure S3).

**Figure 2:**
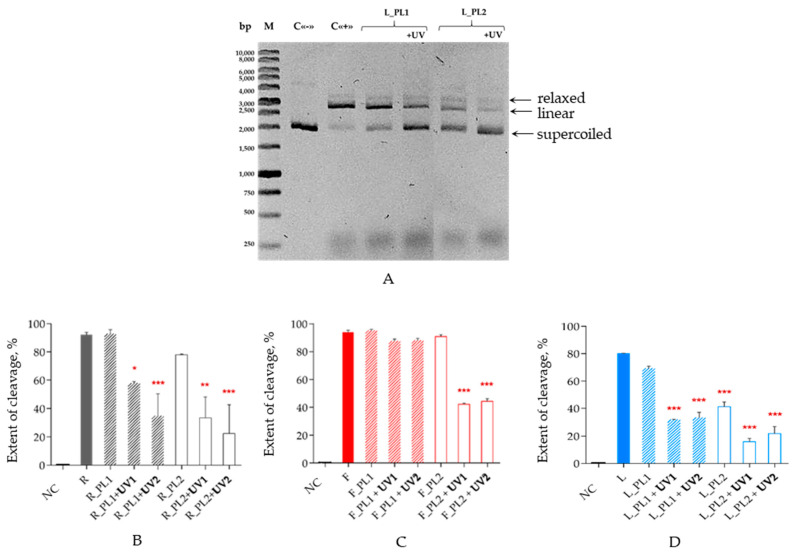
(**A**) Image of a representative agarose gel showing products of cleavage of a protospacer-containing plasmid by Cas9 bound to tracrRNA and L_PL1 or L_PL2 crRNA with or without UV irradiation. M, linear DNA size markers; C«−», no enzyme; C«+», Cas9 bound to non-irradiated native crRNA/tracrRNA. Arrows mark the mobility of the supercoiled, relaxed and linear forms of the plasmid. (**B**–**D**) extent of the plasmid DNA cleavage by Cas9 targeted by photocleavable crRNAs ((**B**) native; (**C**) 2′-fluoro-modified; (**D**) LNA-modified) and non-modified tracrRNA. The crRNAs were irradiated either prior to the complex assembly (UV1) or as part of Cas9 ribonucleoprotein (UV2). NC, negative control (plasmid without any treatment). Mean ± SD is shown (*n* = 3); *, *p* < 0.01; **, *p* < 0.001; ***, *p* < 0.0001 (one-way ANOVA) in comparison with non-photocleavable crRNAs of the same series (R, F or L). Schemes of the substrates, crRNA and tracrRNA, are presented in Supplementary Figure S3.

Since Cas9 is a very low-turnover enzyme, it is usually taken in a large excess over the substrate in in vitro experiments [38]. The reaction was carried out at 37 °C for 1 h at a 50-fold excess of the Cas9/RNA complex over the target, and the products of the DNA cleavage were analyzed by agarose gel electrophoresis. Upon cleavage by Cas9, a supercoiled circular plasmid was converted into the linear form, which migrated slower in the gel. In some cases, we also detected small amounts of the relaxed form of the plasmid, which corresponded to the cleavage of only one strand (Figure 2A and Figure S4). The cleavage extent was calculated as a ratio of the linear plasmid to all plasmid forms.

The introduction of one PL moiety into crRNA barely affected DNA cleavage by itself: only in the case of LNA-modified crRNA (L_PL1) did we register a decrease of ~14% in the cleavage extent (Figure 2D). Two PLs only slightly influenced the cleavage guided by native (R_PL2, 15% decrease; Figure 2B) or 2′-F-modified crRNA (F_PL2, 3%; Figure 2C) but suppressed the cleavage twofold in the case of LNA-modified crRNA (L_PL2). After UV irradiation, both for isolated crRNA and the Cas9/crRNA/tracrRNA complex, in most cases, we observed a decrease in the cleavage efficiency. However, the system containing F_PL1 retained the same level of DNA cleavage after irradiation, which might be due to increased duplex stability imparted by the 2′-fluoro substitution [39].

Two photolinkers provided more prominent inactivation as compared to one PL in all studied variants of crRNA. The systems with LNA-modified crRNA demonstrated the lowest DNA cleavage under the conditions used. In the case of the native photocleavable crRNA, the cleavage extent depended on the mode of irradiation (crRNA alone or in the ribonucleoprotein complex), in contrast to 2′-modified crRNA. The UV irradiation of the R-series crRNA in a complex with tracrRNA and Cas9 resulted in a 1.5–1.7-fold more efficient enzyme inactivation.

### 2.4. The Influence of Photolabile Linkers on DNA Cleavage Specificity

**Figure 3:**
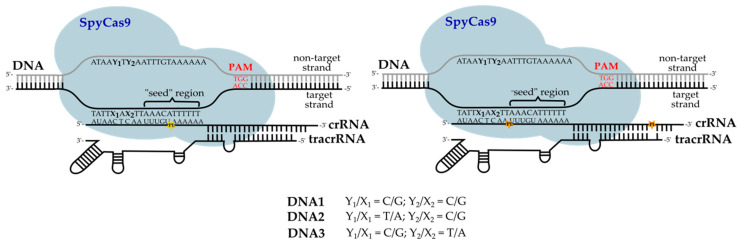
Schemes of model DNA duplexes (DNA1, DNA2, DNA3) bound to Cas9 with PL-containing crRNAs. Y_1_/X_1_ and Y_2_/X_2_ indicate the positions of the replaced base pairs in DNA2 and DNA3.

The specificity of DNA cleavage with the Cas9 guided by different types of crRNA was investigated using three model 50-nt DNA duplexes (Figure 3, Table S2). Each contained a 20-nt protospacer sequence (modified after Psp2) and a PAM. The sequences were designed either to be fully complementary to crRNA (DNA1) or to introduce a single A:C mismatch in positions 5 (DNA2) or 7 (DNA3) of the non-seed region. The cleavage was carried out without any irradiation at different ratios of the effector Cas9 complex to the DNA target.

**Figure 4:**
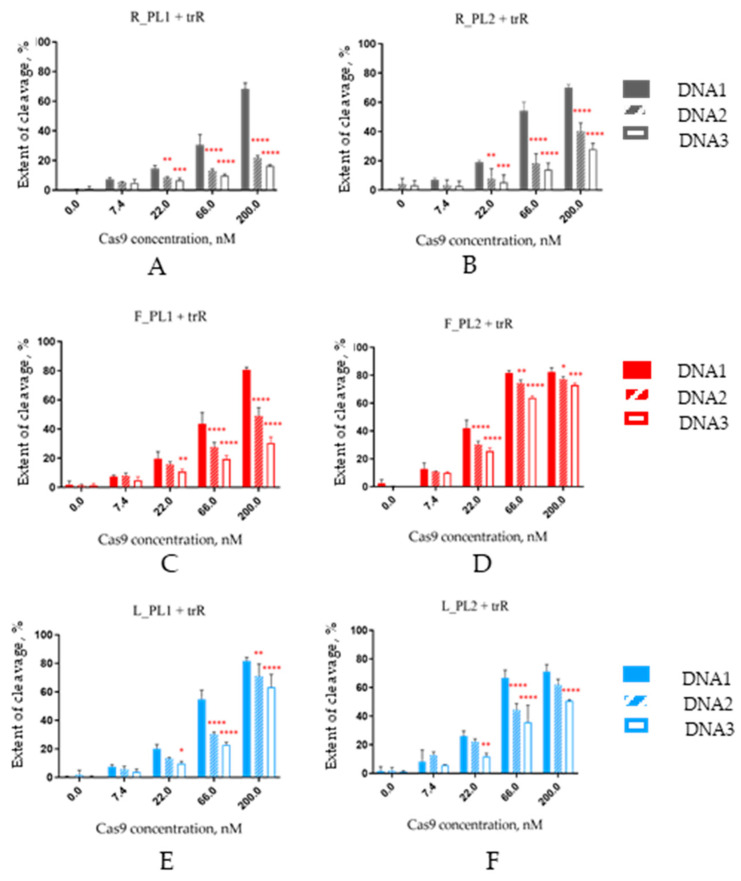
The extent of DNA duplex cleavage by nuclease Cas9 targeted by photocleavable native (**A**,**B**), 2′-fluoro-modified (**C**,**D**) and LNA-modified (**E**,**F**) crRNA with one (**A**,**C**,**E**) or two (**B**,**D**,**F**) photolabile linkers. The specificity of DNA cleavage was investigated using three model 50-nt DNA duplexes labeled by Cy5 fluorescent dye attached to the 3′-end of the non-target strand (Figure 3, Table S2), containing a 20-nt protospacer sequence (Psp2 without mismatches or with a single mismatch) and a PAM. DNA1, Psp2 fully complementary to crRNA; DNA2, a target with a single A:C mismatch in position 5; DNA3, a target with a single A:C mismatch in position 7. The nature of the DNA duplex is indicated in the figures. Mean ± SD is shown (*n* = 3); *, *p* < 0.05; **, *p* < 0.01; ***, *p* < 0.001; ****, *p* < 0.0001 (two-way ANOVA) in comparison with the cleavage of DNA1.

DNA duplexes were labeled by Cy5 fluorescent dye attached to the 3′-end of the non-target strand. After the cleavage, the reaction products were analyzed by denaturing PAGE with fluorescent visualization (Figure S5). The results of the experiment are presented in Figure 4.

When placed in non-modified crRNA and its 2′-fluoro-modified analogs, one PL moiety provided better discrimination between fully complementary DNA1 target and mismatched DNA2 and DNA3 in comparison with two PLs (Figure 4A–D). A rise in Cas9 concentration from 22 nM to 200 nM increased the substrate preference for DNA1 over DNA2 and DNA3 from 1.7–2.2-fold to 3.2–4.2-fold in the case of R_PL1 and from 1.2–1.8-fold to 1.6–2.6-fold in the case of F_PL1. The same trend was also observed for systems with LNA-modified crRNAs, but the discrimination of mismatched DNA targets was less pronounced (Figure 4E,F). The highest preference for the fully complementary target was achieved at a 66 nM Cas9 concentration (1.8–2.4-fold for L_PL1, 1.5–1.9-fold for L_PL2). In all cases, the cleavage of DNA3 was lower in comparison with DNA2. This difference probably originates from the mismatch within the crRNA/DNA3 heteroduplex being closer to the seed region that is most sensitive to incorrect base pairing.

## 3. Discussion

The approach developed in our work makes use of photolabile linkers in the oligonucleotide chain of the crRNA, a molecule that targets Cas9 nuclease to its intended site of action. Photolabile linkers differ by their nature and structure. In particular, those based on 1-(2-nitrophenol) or coumarin photoreactive groups are often used to design photocleavable oligonucleotide constructions [18,40]. In our earlier studies, we developed several types of photoactivatable guide RNAs containing the 1-(2-nitrophenyl)-1,2-ethanediol linker [33,34]. On the other hand, we and others have demonstrated that CRISPR/Cas9 systems containing other modifications in specific positions of the RNA backbone, such as 2′-fluoro or LNA, are more sensitive to single nucleotide mismatches within a DNA target [32,41,42]. An additional advantage of 2′-modified crRNA is their higher stability with regard to the non-specific degradation by nucleases present in biological fluids [32]. Herein, we combined these two approaches to explore a way towards more efficient photocleavable gRNAs that can be used for controlled inactivation of the CRISPR/Cas9 systems.

Photocleavable crRNAs designed and investigated in this study differ by the presence of a 2′-modification (RNA, 2′-F-RNA and LNA-modified RNA), location and number of photolabile linkers. 2′-F-RNAs contained 2′-fluoropyrimidine nucleotides in certain positions of the crRNA that are not involved in interactions with the Cas9 protein [15,43]. In LNA-modified RNAs, several uridine ribonucleotides were replaced by LNA-thymidines to improve RNA nuclease stability and affinity for the complementary DNA target [42,44].

Upon irradiation at 365 nm (UVA range), photolabile linkers-containing crRNA were subjected to cleavage, yielding two (one PL) or three (two PL) fragments. The 30 min irradiation time was used to ensure full degradation of all crRNA constructs, but in practice, it likely could be shortened since the half-lives of all constructs, with the exception of L_PL1, were under 2 min, which translates into >100-fold reduction in the amount of full-length crRNA in less than 15 min. Of note, UVA irradiation at >350 nm is much less damaging to living cells than shorter-wavelength UVA (315–350 nm), UVB or UVC [45], which is important for the possible practical applications of photoinactivatable CRISPR/Cas9 systems. Photocaged oligonucleotides and other biomolecules converted by near-UV irradiation have been used in numerous studies in living cells and multicellular organisms with no notable side effects [46,47]. 2′-Modified crRNAs with two PLs were degraded faster than those with one PL. The modifications also affected the photodegradation rate: both 2′-F- and LNA-modified RNAs were less sensitive to UV irradiation than non-modified photocleavable RNA (Table 1). This effect could be explained by the influence of the secondary structure stability and possible differences in the reaction quantum yield due to stacking or hydrophobic interactions of the aromatic nitrobenzyl moiety with the adjacent modified nucleotides.

We employed two model systems with different DNA targets to assess the activity of Cas9 targeted by the designed photocleavable crRNA and non-modified tracrRNA. To estimate the effect of crRNA cleavage on the CRISPR/Cas9 system, we employed a plasmid substrate containing a Psp2 protospacer targeted by crRNA, together with the PAM responsible for target recognition. The Psp2 sequence comes from a spacer identified in the *S. pyogenes* genome in the seminal work of Charpentier, Doudna and colleagues [7] and has been widely used as a reference Cas9 substrate ever since [38,48,49,50]. Off-target effects were studied with model fluorescently labeled DNA duplexes derived from the same sequence, either fully complementary to the corresponding crRNA fragment or containing single nucleotide mismatches within the non-seed region.

In the absence of UV irradiation, one PL moiety introduced into crRNA did not affect the extent of plasmid cleavage by Cas9 (Figure 2). Two PLs within the 2′-F-modified crRNA (F_PL2) also did not change the efficiency of DNA cleavage. On the contrary, in the cases of non-modified crRNA (R_PL2) or its LNA-modified analog (L_PL2), the cleavage decreased by 15% and 50%, respectively. We then assessed two variants of crRNA inactivation before DNA cleavage: crRNA was irradiated either separately or within the fully assembled complex with tracrRNA and Cas9 nuclease. Since crRNA would presumably be retained in the complex after the cleavage, we expected to see differences between these two modes but, in fact, observed no effect, with a marginally lower (~1.5–1.7-fold) nuclease activity only in the case of the non-modified photocleavable crRNA (R_PL1 and R_PL2) in a complex as compared to the irradiation of isolated crRNA, and an equally unremarkable increase in a complex vs. isolated crRNA for L_PL2. This could mean that Cas9 can bind crRNA which is split into two or three pieces, of which one is complementary to the anti-repeat part of tracrRNA. This possibility is worth further investigation.

We also inquired whether PLs within crRNA could aggravate the off-target effects of editing without irradiation. As we demonstrated previously, 2′-F and LNA-modified crRNAs are more sensitive to nucleotide changes in the target DNA. We, therefore, hypothesized that in biological systems, their off-target effects would be less prominent than for unmodified crRNA [32]. In this present study, we estimated the contribution of photolabile linkers to off-target cleavage. Rather unexpectedly, non-modified photocleavable crRNAs demonstrated the most pronounced specificity of DNA cleavage in vitro in contrast to 2′-F-RNA (F_PL1 and F_PL2) and LNA-modified RNA (L_PL1 and L_PL2; Figure 4). Non-modified PL-containing crRNAs provided maximal difference (3.1–4.2-fold) between the cleavage extent of the fully complementary DNA target (DNA1) and two targets containing mismatches (DNA2 and DNA3). We proposed that PL insertions may partially destabilize the DNA/RNA heteroduplex while 2′-modified RNAs have the opposite effect, and thus, even complexes with mismatched targets are tight enough for sufficient cleavage with 2′-modified guides.

It is noteworthy that one PL within crRNA induced less off-target cleavage than two PLs. One PL was introduced into the seed region of the protospacer, and this position is more sensitive to local disruptions. We assume that the selection of optimal positions for the introduction of one or several PLs could permit the creation of a photoregulatable CRISPR/Cas9 system with minimal off-target effects.

## 4. Materials and Methods

### 4.1. Chemicals, Enzymes and Plasmids

A controlled pore glass (CPG) support bearing the first nucleoside, amino-modified CPG support, 5′,*N*-protected 2′-*O*-TBDMS-ribo (A, C, G or U) and deoxyribo (dA, dC, dG or dT) nucleoside phosphoramidites were purchased from Glen Research (Sterling, VA, USA). 5′,*N*-protected 2′-fluoro-deoxyribo (C and U) was from ChemGene (Wilmington, MA, USA) and LNA T phosphoramidite, from MilliporeSigma (Burlington, MA, USA). All organic solvents (THF, CH_3_CN, ethanol, CH_2_Cl_2_) were dried by 3-Å molecular sieves or by distillation and stored over CaH_2_. Recombinant Cas9 endonuclease and pBS2SKM Psp2 plasmid were obtained according to the published protocols [38]. The Cas9/crRNA/tracrRNA complex was assembled by adding Cas9 protein (1.35 pmol) to a preliminary annealed crRNA/tracrRNA duplex (1.35 pmol) in the cleavage buffer containing 20 mM HEPES–KOH (pH 7.5), 100 mM KCl, 2 mM MgCl_2_, 1 mM DTT, 0.5 mM Na_2_EDTA, and 5% glycerol. The mixture was vortexed, kept for 15 min at 37 °C, and used immediately.

### 4.2. Oligonucleotides

Oligonucleotide synthesis was carried out on an ASM-800 DNA/RNA synthesizer (Biosset, Novosibirsk, Russia) using the protocols optimized for this instrument. Amino-modified polymer 3′-PT-Amino-Modifier C6 CPG (Glen Research) was used for the synthesis of 3′-amino-modified DNA. A 3% solution of dichloroacetic acid in dichloromethane was used for the detritylation step. Propionic anhydride and methylimidazole were used for capping. Iodine solution in tetrahydrofuran:H_2_O:pyridine mixture was used for oxidation. The synthesis was carried out with 0.1 M solutions of ribonucleotide phosphoramidites or 0.05 M solutions of deoxyribonucleotide phosphoramidites in absolute acetonitrile. For RNA synthesis, a 0.25 M solution of activator 42 (MilliporeSigma) in absolute acetonitrile was used at the condensation step; for DNA synthesis, we used a 0.25 M solution of 5-ethylthiotetrazole in absolute acetonitrile. The time of condensation was 7 min for RNA and 4 min for DNA. The last 5′-*O*-dimetoxytrityl group was removed, and oligonucleotides were deprotected under the standard conditions: 40% aqueous methylamine for 15 min at 65 °C (DNA oligonucleotides) or AMA (40% methylamine, 30% ammonium, 1:1) for 2 h at room temperature with subsequent treatment with NMP:TEA:TEA·3HF mixture for 1.5 h at 65 °C, addition of ethoxytrimethylsilane and precipitation with ethyl ether (RNA oligonucleotides). All oligonucleotides were purified by denaturating PAGE.

### 4.3. Synthesis of Photocleavable crRNA

Phosphoramidite of 1-(2-nitrophenyl)-1,2-ethanediol was synthesized as described in [33,34,35]. We used a 0.1 M solution of this phosphoramidite and a 0.25 M solution of activator 42 in absolute acetonitrile at the condensation step, which was extended to 30 min. The deprotection conditions were the same as for unmodified RNA.

### 4.4. Preparation of Cy5-Labeled DNA Duplexes

N-oxysuccinimide ether of cyanine 5 (Cy5 NHS) (Lumiprobe, Moscow, Russia) was used for 3′-amino-modified DNA labeling. A freshly prepared solution of Cy5 NHS in dimethylsulfoxide was added to the solution of 3′-amino modified DNA in 0.05 M Tris–HCl (pH 8.3). The reaction mixture was incubated at 37 °C while stirring for 2 h, then the oligonucleotide material was precipitated by 2% NaClO_4_ in acetone. Cy5-labeled oligonucleotides were isolated by denaturating PAGE and annealed to the complementary strands in 10 mM HEPES–KOH (pH 7.5) by heating to 95 °C and slowly cooling to room temperature. Then, casein solution was added up to 1 mg/mL to prevent adsorption on tube walls during storage. The duplexes were stored at −20 °C.

### 4.5. Cleavage of crRNA by UV Irradiation

Solutions of 0.05 OD_260_ of crRNA carrying a PL moiety in 10 µL of deionized water were placed in clear 0.5-mL polypropylene tubes transparent in the UVA range (Eppendorf, Hamburg, Germany) and irradiated at room temperature with a light of a 365 nm wavelength using a TCP-20.LC UV transilluminator (Vilber Lourmat, Marne-la-Vallée, France) equipped with five 8-W 365 nm light tubes. After 10 s, 30 s, 1 min, 5 min and 30 min, probes were placed in the dark and then analyzed by reverse-phase HPLC on an Alfachrom chromatograph (Econova, Novosibirsk, Russia) equipped with a ProntoSil-120-5-C18 column (75 mm × 2 mm) in a 0–50% acetonitrile gradient in 0.02 M triethylammonium acetate (pH 7.0) run for 20 min at a 100 µL/min flow rate. Absorbance was registered at 260, 280 and 300 nm. The data were processed using the Multichrom software package version 2.4 (Ampersand Ltd, Moscow, Russia). The fraction of intact RNA was calculated as a ratio of the area of its peak to the sum of the areas of all peaks in the profile. The rate constant of photocleavage *k* was determined by numerical fitting in the GraphPad Prism v7.0 software (GraphPad Software, Boston, MA, USA) using the equation:

(1) $${RNA}_{t}=\left({RNA}_{0}-{RNA}_{\infty }\right)e^{-kt}+{RNA}_{\infty }$$

where RNA*_t_* is the remaining fraction of intact RNA at time *t*, RNA_0_ is the fraction of intact RNA at zero time, and RNA_∞_ is the remaining fraction of intact RNA at infinite time.

In the plasmid cleavage experiments (see below), the irradiation was carried out before the cleavage in two variants: (1) crRNA was irradiated separately at 365 nm for 30 min, then assembled into a complex with tracrRNA and Cas9 protein); (2) the crRNA:tracrRNA:Cas9 complex was assembled and then irradiated at 365 nm for 30 min.

### 4.6. Plasmid Digestion by CRISPR/Cas9

Plasmid cleavage reactions were carried out as described in [32]. Briefly, a 50-fold molar excess of the effector complex was added to 50 ng of the plasmid and incubated for 1 h at 37 °C in 10 μL of a buffer containing 20 mM HEPES–KOH (pH 7.5), 100 mM KCl, 2 mM MgCl_2_, 1 mM DTT, 0.5 mM Na_2_EDTA, and 5% glycerol. To stop the reaction, 2.5 µL of quenching solution (250 mM Na_2_EDTA, 1.2% SDS, 0.01% bromophenol blue in 30% glycerol) was added. Cleavage products were resolved by 1% agarose gel electrophoresis with ethidium bromide staining, visualized using the E-Box-CX5 gel documentation system (Vilber Lourmat) and quantified with Quantity One v4.6.8 software (Bio-Rad Laboratories, Hercules, CA, USA). The fraction of cleaved plasmid (*N*) was calculated as follows:

(2) $$N=\frac{I_{III}}{{1.14\times I}_{I}+I_{II}+I_{III}}$$

where *I*_I_, *I*_II_ and *I*_III_ are the intensities of the bands corresponding to plasmid forms I (supercoiled), II (relaxed) and III (linear), respectively, and the coefficient 1.14 accounts for different binding of ethidium bromide to the supercoiled plasmid [51].

### 4.7. Cleavage of Model DNA Duplexes by CRISPR/Cas9

The reaction mixture (10 µL) included 20 mM HEPES–KOH (pH 7.5), 100 mM KCl, 10 mM MgCl_2_, 1 mM DTT, 5% glycerol, 0.2 mg/mL poly(A), 100 nM substrate oligonucleotide duplex, and 7.4 nM, 22 nM, 66 nM, or 200 nM Cas9/crRNA/tracrRNA effector complex. The reaction was allowed to proceed for 1 h at 37 °C and was stopped by adding 20 µL of the solution containing 5 mM Na_2_EDTA (pH 7,5), 0.1 mg/mL Orange G, 0.025% SDS and 5% glycerol in 90% formamide. The cleavage products were resolved by 12% denaturing PAGE, visualized using a Typhoon FLA 9500 imager (GE Healthcare, Chicago, IL, USA) with the following parameters: Cy5 dye, 600 V, 50 μm resolution, and quantified with Quantity One v4.6.8 software.

### 4.8. Statistical Treatment

Each experiment was repeated at least three times. Statistical analysis was carried out using GraphPad Prism 7.00 software (GraphPad). A one-way or two-way ANOVA with Dunnett’s correction for multiple comparisons was used to estimate the significance of the differences, as indicated in figure legends. The differences were considered significant at *p* < 0.05.

## 5. Conclusions

The photocleavable guide crRNAs and their 2′-modified (2′-fluoro and LNA) analogs developed in the present work represent controllable components of the CRISPR/Cas9 system. One or two linkers of the 1-(2-nitrophenyl)-1,2-ethanediol nature introduced into the oligonucleotide strand allowed crRNA fragmentation to be turned on in a spatially and temporally controlled manner, thus switching the CRISPR/Cas9 system off. We also thoroughly investigated the effects of PLs on crRNA functional properties before irradiation. Prior to irradiation, crRNA with two photolabile linkers retains its ability to induce complete and fast degradation of a DNA target. Moreover, either one or two PLs within a non-irradiated crRNA structure even improved the specificity of DNA cleavage. The minimal off-target effect (up to a 4.2-fold decrease in the cleavage of mismatched DNA) was demonstrated for non-modified crRNA with a single PL. Therefore, the location of PL in gRNA is of utmost importance for the specificity of DNA cleavage by Cas9. These results require further testing on other DNA sequences and the selection of optimal positions and the number of photolabile linkers.

To summarize, the use of photocleavable crRNA developed in this work as an addressing component in CRISPR/Cas9 systems provides the possibility of spatiotemporal control of this system with reduced off-target effects. Controllable and precise CRISPR/Cas9 systems are promising tools for modern molecular biology and genetic technology. The approach proposed herein seems to be amenable to further improvements, including optimization of the type and position of the modifications, and the number and location of photolabile linkers for DNA targets of interest.

## Figures and Tables

**Table 1:** Photocleavage rate constants and half-lives of crRNA with a photolabile linker.

	R_PL1	R_PL2	F_PL1	F_PL2	L_PL1	L_PL2
*k*, min^−1^	0.72 ± 0.11	0.58 ± 0.09	0.35 ± 0.10	0.77 ± 0.07	0.24 ± 0.10	0.54 ± 0.17
*t*_1/2_, min	0.96	1.20	1.99	0.90	2.85	1.27

## Data Availability

Data is contained within the article and Supplementary Materials.

## Supplementary Materials

The following supporting information can be downloaded at: https://www.mdpi.com/article/10.3390/ijms252212392/s1.
