# Supplementary material for: Photocleavable Guide crRNAs for a Light-Controllable CRISPR/Cas9 System
Source: Int J Mol Sci. 2024 Nov 19;25(22):12392. doi: 10.3390/ijms252212392 (PMC11594570; doi:10.3390/ijms252212392)
Supplement: Supplementary file 1 [file ijms-25-12392-s001.zip › ijms-3286910-supplementary.pdf]

Supplementary Information

Photocleavable Guide crRNAs for a Light-Controllable CRISPR/Cas9 System

Lubov Sakovina [1,2], Ivan Vokhtantsev [1,2], Elizaveta Akhmetova [1,2], Mariya Vorobyeva [1], Pavel Vorobjev [1], Dmitry O. Zharkov [1,2] and Darya Novopashina [1,2,*]

[1] Institute of Chemical Biology and Fundamental Medicine SB RAS, 630090 Novosibirsk, Russia;
kodi99@list.ru (L.S.); ivanvohtancev@gmail.com (I.V.); liza.khabardina@mail.ru (E.A.); kuzn@niboch.nsc.ru (M.V.); vorobyev@niboch.nsc.ru (P.V.); dzharkov@niboch.nsc.ru (D.O.Z.)

[2] Faculty of Natural Sciences, Novosibirsk State University, 630090 Novosibirsk, Russia

* Correspondence: danov@niboch.nsc.ru

Synthesis of 2′-modified photocleavable and native guide RNA

The sequences of 2′-modified photocleavable and native guide RNA are presented in the Table S1.

Table S1. The sequences of 2'-modified photocleavable and native guide RNA used in this work.

Name	Sequences, 5′→3′ [1]
R	AUAACUCAAUUUGUAAAAAAGUUUUAGGCUAUGCUGUUUUG
R_PL1	AUAACUCAAUUUGU–**PL**–AAAAAAGUUUUAGGCUAUGCUGUUUUG
R_PL2	AUAACUCAA–**PL**–UUUGUAAAAAAGUUUUAGGCUAUGCUG–**PL**–UUUUG
F	AU^{F}AACUCAAU^{F}U^{F}UGU^{F}AAAAAGUUUUAGAGC^{F}U^{F}AU^{F}GC^{F}U^{F}GU^{F}U^{F}U^{F}U^{F}G
F_PL1	AU^{F}AACUCAAU^{F}U^{F}UGU^F–**PL**–AAAAAGUUUUAGAGC^{F}U^{F}AU^{F}GC^{F}U^{F}GU^{F}U^{F}U^{F}U^{F}G
F_PL2	AU^{F}AACUCAA–**PL**–U^{F}U^{F}UGU^{F}AAAAAGUUUUAGAGC^{F}U^{F}AU^{F}GC^{F}U^{F}G–**PL**–U^{F}U^{F}U^{F}U^{F}G
L	AT^{L}AACUCAAUT^{L}UGT^{L}AAAAAAGUUUUAGAGCT^{L}AUGCT^{L}GUT^{L}UUG
L_PL1	AT^{L}AACUCAAUT^{L}UGT^L–**PL**–AAAAAAGUUUUAGAGCT^{L}AUGCT^{L}GUT^{L}UUG
L_PL2	AT^{L}AACUCAA–**PL**–UT^{L}UGT^{L}AAAAAAGUUUUAGAGCT^{L}AUGCT^{L}G–**PL**–UT^{L}UUG
trR	AACAGCAUAGCAAGUUAAAAUAAGGCUAGUCCGUUAUCAACUUGAAAAAGUGGCACCGAGUCGGUGCUUUUUUU

[1] The fragment of crRNA complementary to the DNA protospacer is underlined; **N^F**, 2′-fluoro pyrimidine nucleotide; **T^L**, LNA-thymidine; **PL**, photolabile 1-(2-nitrophenyl)-1,2-ethanediol linker.

Photocleavable crRNA degradation upon UV irradiation

All synthesized RNAs were purified by 15% denaturing polyacrylamide gel electrophoresis (PAGE), and their homogeneity was analyzed by reverse-phase HPLC and denaturating PAGE (Figure S1).

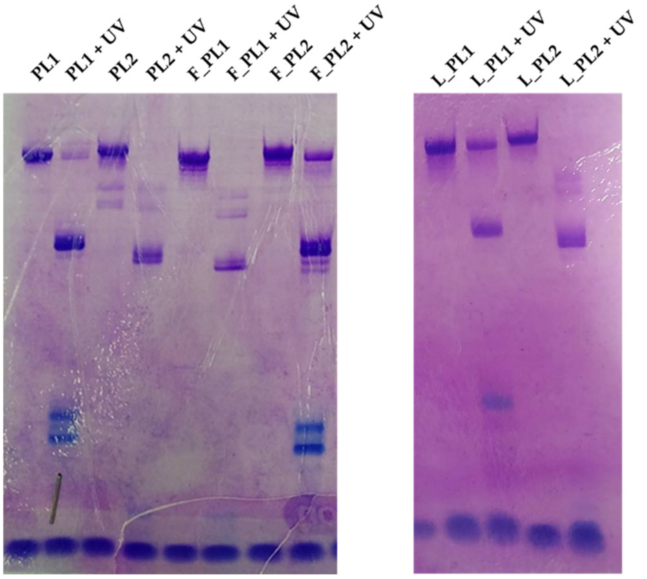

Figure S1. Analysis of photomodified crRNAs and their UV-cleavage products by denaturating 15% PAGE, amout of RNA per line 0.03 AU_{260}, UV wavelength 365 nm, irradiation time 5 min. The gel was stained by Stains-all dye. The bands at the bottom are bromephenol blue.

Water solutions of crRNA were irradiated with the 365 nm UV light up to 30 min, aliquots were withdrawn after 0, 1, 5 and 30 min and analyzed by reverse-phase HPLC. Disappearance of the peaks corresponding to initial photomodified crRNA and appearance of peaks with lower retention times corresponding to the products of photodegradation was observed (Figure S2).

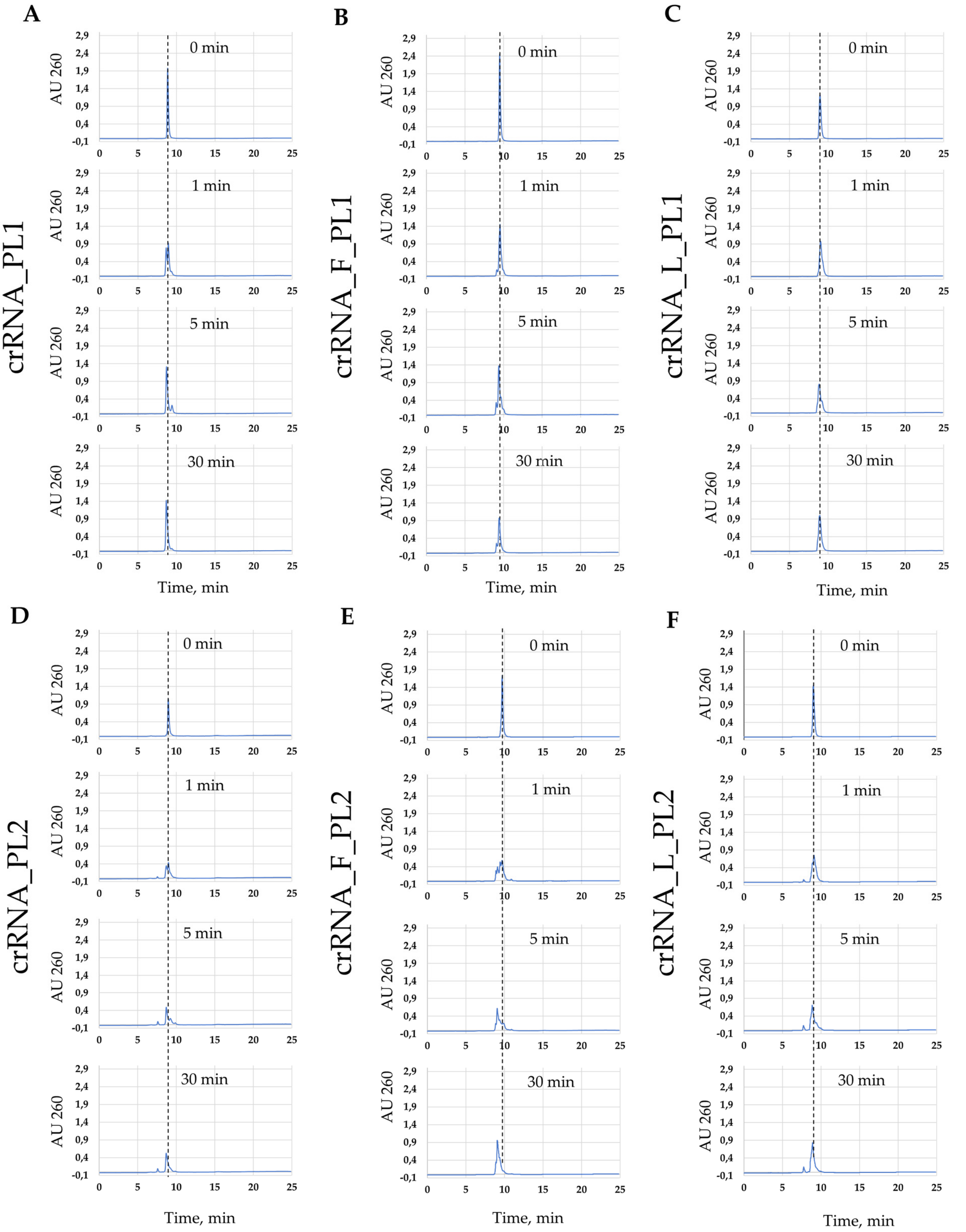

Figure S2. Reverse-phase HPLC profiles of UV cleavage products of photomodified crRNAs in 0–50% acetonitrile gradient in 0.02 M triethylammonium acetate (pH 7.0): crRNA_PL1 (A), crRNA_F_PL1 (B), crRNA_L_PL1 (C) crRNA_PL2 (D) crRNA_F_PL2 (E) crRNA_L_PL2 (F); UV absorption at 260 nm followed. Cleavage conditions: 0.05 AU_{260} of photomodified crRNA in 10 μl of Milli-Q quality water, UV irradiation at 365 nm for 0, 1, 5 and 30 min.

Cleavage of plasmid DNA by Cas9 in a complex with photocleavable crRNA and non-modified tracrRNA

DNA cleavage was carried out by the Cas9 effector complex with photomodified crRNAs that were non-irradiated (Figure S3A), irradiated separately before effector complex formation (Figure S3B) or irradiated after the effector complex assembly (Figure S3C). Irradiation was carried out by 365-nm UV light for 30 min.

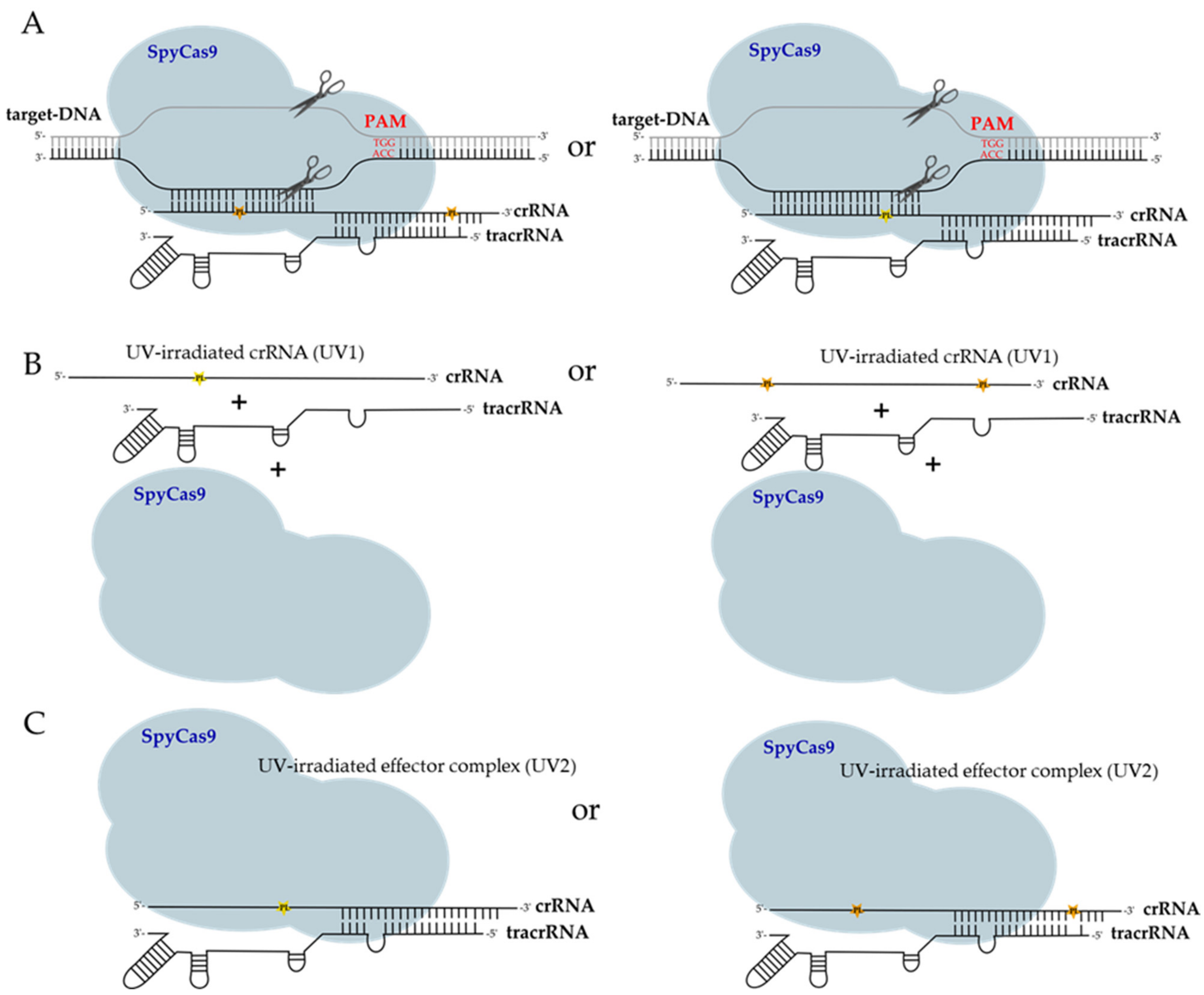

Figure S3. Schematic representation of experimental design for the investigation of the influence of one or two PLs in the crRNA structure on Cas9 activity. (A) Complex of the DNA target with components of CRISPR/Cas9 system. (B) UV irradiation (UV1) of individual crRNA containing one (yellow star) or two (orange stars) PLs. (C) UV irradiation (UV2) of crRNA in the effector complex with Cas9 protein. PAM, protospacer adjacent motif.

Plasmid cleavage was carried out at a 50:50:1 Cas9:crRNA+tracrPHK:plasmid DNA ratio in a buffer contaning 20 mM HEPES–KOH (pH 7.5), 100 mM KCl, 1 mM DTT, 0.5 mM Na_2EDTA, 2 mM $MgCl_2$, 5% glycerol for 1 h at 37°C. Analysis of the products was performed by electrophoresis in 1% agarose gel in Tris–acetate–EDTA buffer with ethidium bromide staining (Figure S4). Upon cleavage of the supercoiled plasmid, a linear form with a lower mobility appeared. Also, small amount of the relaxed plasmid was detected in some cases.

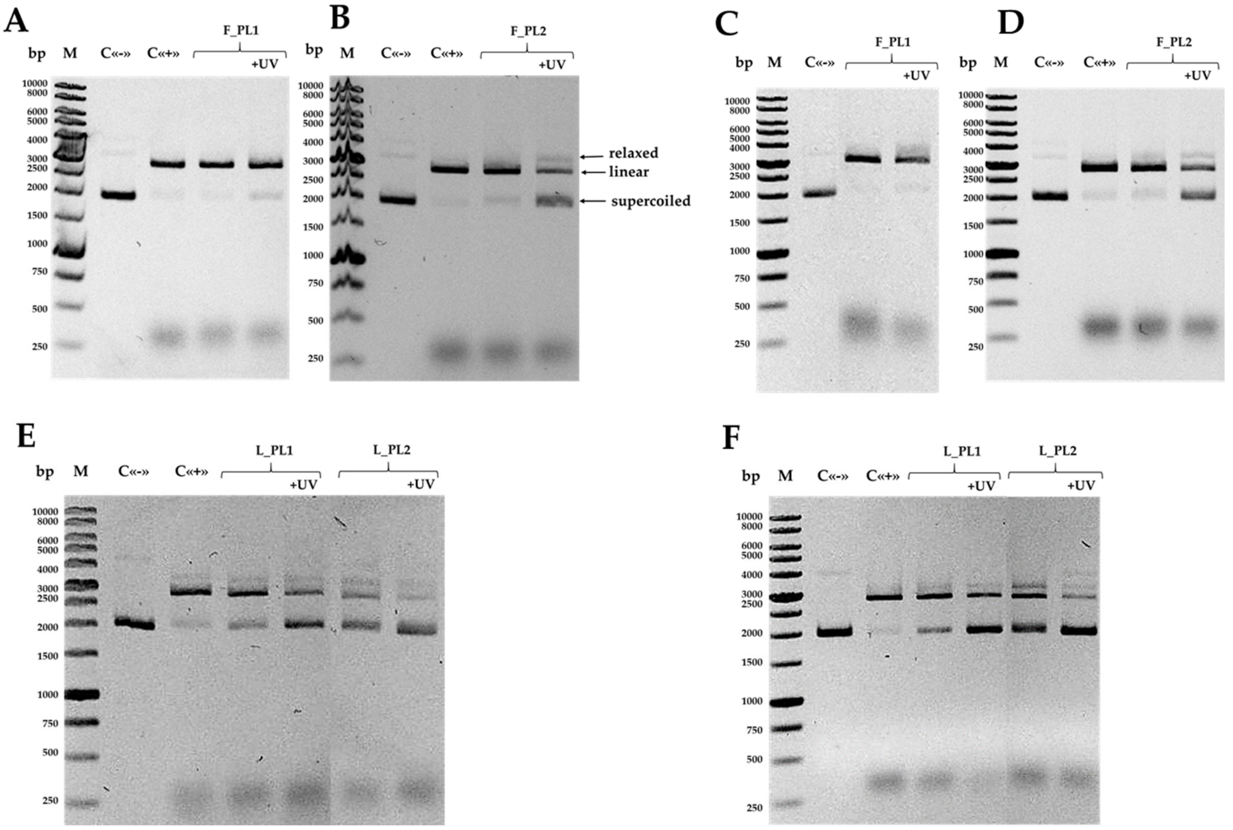

Figure S4. Analysis of plasmid cleavage products by 1% agarose gel electrophoresis. The Cas9 effector complex was assembled with pre-irraduated crRNA: (A) crRNA_F_PL1, (B) crRNA_F_PL2, (E) crRNA_L_PL1, crRNA_L_PL1, or the pre-assembled complex was irradiated: (C) crRNA_F_PL1, (D) crRNA_F_PL2, (F) crRNA_L_PL1, crRNA_L_PL1. M, DNA size markers; C«-», plasmid alone, C«+», plasmid treated with the Cas9 effector complex with non-modified crRNA and tracrRNA.

The influence of photolabile linkers on DNA cleavage specificity

The specificity of DNA cleavage was investigated using three model 50-nt DNA duplexes (Table S2). The duplexes contained a protospacer sequence, PAM and a Cy5-label on the 3′-end of the non-target strand. The protospacers differed by one nucleotide pair in distal parts of protospacer. DNA1 contained the sequence fully complementary to crRNA and DNA2 and DNA3 contained G:C>T:A substitutions in 5th or 7th positions of the protospacer, respectively. The cleavage was carried out without irradiation at different ratios of the effector Cas9 complex to DNA target. The reaction products were analyzed by denaturing PAGE electrophoresis with fluorescent visualization (Figure S5).

Table S2. Sequences of model DNA duplexes components*.

Name	Sequences
DNA1	Non-target strand:<br>5′-TTATATGAAGATAACTCAATTTGTAAAAAA**TGG**TATTGGGGAATTCATTA-**Cy5**-3′<br>Target strand:<br>3′-AATATACTTC**TATTGAGTTAAACATTTTTT**ACCATAACCCCTTAAGTAAT-5′
DNA2	Non-target strand:<br>5′-TTATATGAAGATAA**T**TCAATTTGTAAAAAA**TGG**TATTGGGGAATTCATTA-**Cy5**-3′<br>Target strand:<br>3′-AATATACTTC**TATTAAGTTAAACATTTTTT**ACCATAACCCCTTAAGTAAT-5′
DNA3	Non-target strand:<br>5′-TTATATGAAGATAACT**T**AATTTGTAAAAAA**TGG**TATTGGGGAATTCATTA-**Cy5**-3′<br>Target strand:<br>3′-AATATACTTC**TATTGAATTAAACATTTTTT**ACCATAACCCCTTAAGTAAT-5′

* **PAM** is marfked with in bold red symbols, **protospacer**, with bold black symbols, **nucleotide replacements**, with bold blue symbols.

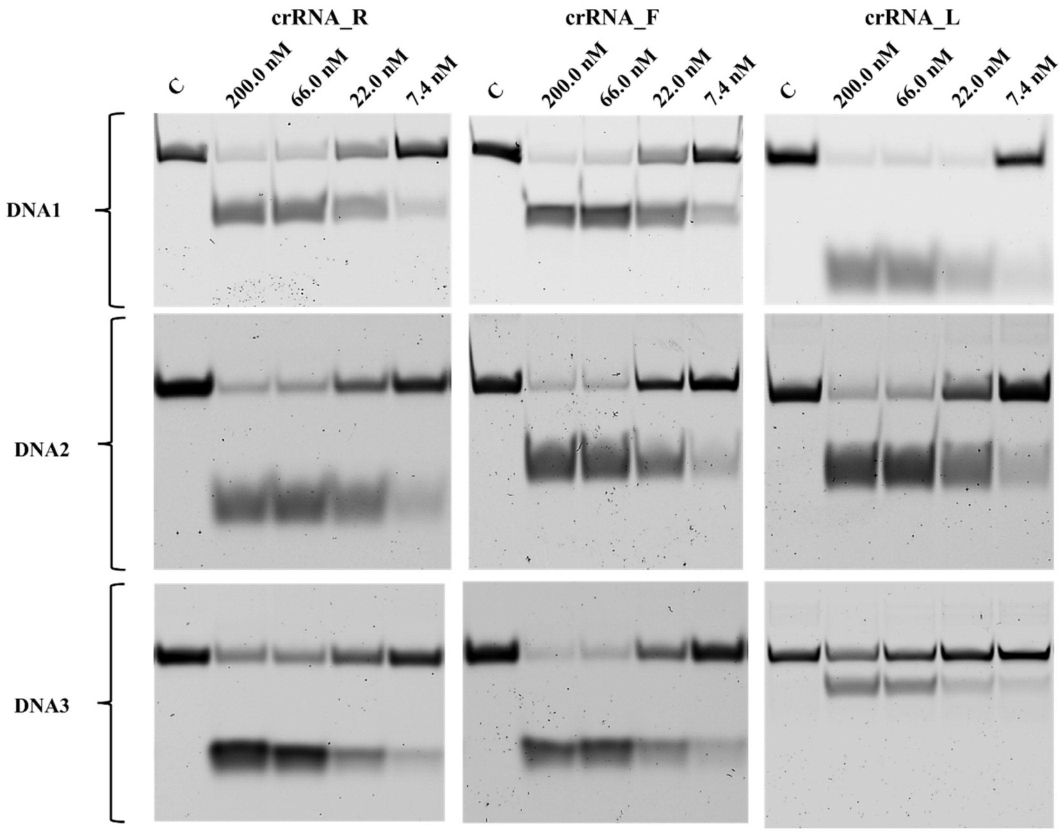

Figure S5. Analysis of Cy5-labelled dsDNA cleavage products by 12% denaturing PAGE. 200.0 nM, 66.0 nM, 22.0 nM, 7.4 nM are the concentrations of Cas9 effector complex. C, DNA duplex in absence of the effector complex. Cleavage conditions were 20 mM HEPES–KOH (pH 7.5), 100 mM KCl, 1 mM DTT, 10 mM $MgCl_2$, 5 % glycerol, 0.2 mg/ml PolyA, 10 nM substrate DNA duplex, 37 °C, 1 h.
